# Quantitative changes in iris and retinal blood flow after femtosecond laser-assisted *in situ* keratomileusis and small-incision lenticule extraction

**DOI:** 10.3389/fmed.2022.862195

**Published:** 2022-08-05

**Authors:** Lipu Cui, Wenwen Xue, Wenbo Yao, Xinhui Huang, Wen Xue, Yulan Wang, Shanshan Li, Liquan Zhao, Haidong Zou

**Affiliations:** ^1^Shanghai Key Laboratory of Ocular Fundus Diseases, Shanghai General Hospital, Shanghai Jiao Tong University School of Medicine, Shanghai Engineering Center for Precise Diagnosis and Treatment of Eye Diseases, National Clinical Research Center for Eye Diseases, Shanghai, China; ^2^Shanghai Eye Diseases Prevention and Treatment Center/Shanghai Eye Hospital, Shanghai, China

**Keywords:** optical coherence tomography angiography, FS-LASIK, SMILE, ocular blood circulation indices, vascular quantification

## Abstract

**Purpose:**

To investigate the quantitative changes in iris and retinal blood flow indices after femtosecond laser-assisted *in situ* keratomileusis (FS-LASIK) and small-incision lenticule extraction (SMILE).

**Methods:**

Seventy-nine patients who underwent FS-LASIK or SMILE were enrolled between July 2020 and September 2020. Participants were followed-up 1 day pre-operatively and 1 week, 1 month, 3 months post-operatively. Optical coherence tomography angiography (OCTA) was used to acquire and quantify the iris and retinal blood flow indices.

**Results:**

The iris vessel area density (VAD) and vessel skeleton density (VSD) decreased on post-operative day 1 but recovered on day 7. In both cases, the pupil diameter was positively associated with the post-operative iris blood flow indices (*p* = 0.0013, *p* = 0.0002). The retinal VAD and VSD in the superficial and deep capillary plexuses decreased after surgery and failed to recover after 90 days. The SMILE group showed significantly lower iris and retinal blood flow indices than the FS-LASIK group. For both procedures, axial length (*p* = 0.0345, *p* = 0.0499), spherical equivalence (*p* = 0.0063, *p* = 0.0070), and suction duration (*p* = 0.0025, *p* = 0.0130) were negatively correlated with the post-operative VAD and VSD.

**Conclusions:**

The SMILE and FS-LASIK procedures induced a short-term decrease in the iris and retinal blood flow indices, although patients finally showed full visual recovery. This phenomenon should be carefully considered, especially in patients prone to anterior segment lesions.

## Introduction

Uncorrected refractive error is the leading cause of visual impairment worldwide (1). The most commonly used surgical procedures for refractive error correction are the femtosecond laser-assisted *in situ* keratomileusis (FS-LASIK) and the small-incision lenticule extraction (SMILE) (2). Although intraoperative injuries are mild for both procedures, many kinds of post-operative complications can occur (3, 4). These include lesions in anterior segment structures such as the iris. In this vein, Kenan et al. reported a case of iris atrophy after FS-LASIK; the iris lesion was consistent with the femtosecond laser trace that originated during the flap creation (5). In theory, the intraoperative femtosecond laser can reach the iris and cause damage to the vasculature, and high intraoperative negative pressure can also affect the blood flow in the iris tissue. Furthermore, FS-LASIK and SMILE are both performed using a negative suction ring to facilitate the creation of a corneal flap or lenticule, which could induce intraocular pressure (IOP) above 65 mmHg (6). However, there has been no research on the changes in iris circulation and possible influencing factors after FS-LASIK and SMILE.

From 2000 to 2001, Cameron et al. reported 6 cases of post-LASIK patients with severe posterior segment complications (6–8). Cameron et al. suggested that intraoperative elevated IOP caused the posterior segment complications (5–8), which could also affect the retinal vasculature. Usually, the suction duration is longer in SMILE than in FS-LASIK, Zhang et al. found macular vessel density decreased after FS-LASIK and recovered 1 month post-operatively (9); however, Shen. et al. discovered a decreased macular vessel density recovered 7 days post-operatively (10). Further studies are needed to compare and analyze the retinal vasculature changes and the underlying factors after FS-LASIK and SMILE.

This prospective cohort study investigated the iris and retinal blood flow changes after FS-LASIK and SMILE using optical coherence tomography angiography (OCTA). Our results provide a novel insight for mitigating post-operative complications.

## Methods

This prospective longitudinal cohort study was registered on the clinical trial website (NCT03631108). The study followed the tenets of the Declaration of Helsinki and was approved by the Ethics Committee of the Shanghai General Hospital, Shanghai Jiao Tong University School of Medicine (2018KY181). Investigators explained the study procedures to all participants and obtained written informed consent. Seventy-nine patients with myopia who planned to undergo corneal refractive surgery at the Shanghai Eye Disease Prevention & Treatment Center/Shanghai Eye Hospital were enrolled between July 2020 and September 2020. The inclusion criteria included: age range 18–45 years old, stable myopia for at least 2 years, best-corrected distance visual acuity of at least 20/20, refractive error <-7.00 diopters (D), spherical error <-3.00 D of astigmatism, and central corneal thickness (CCT) >480 μm. The exclusion criteria included: abnormal corneal topography (K < 39.7 D or K > 51.9 D); corneal opacity affecting laser penetration; obvious iris changes such as tumors, nodules, and neovascularization; history of trauma, surgery, and ocular fundus disease; systemic diseases such as diabetes mellitus and hypertension; and poor quality of OCTA images.

Participants were divided into two groups according to the type of surgery (FS-LASIK or SMILE). The surgeons (S. L. and L. Z.) recommended one procedure based on the patient's corneal thickness and refractive error, respecting the patient's wishes. The SMILE procedure was performed using a 500-kHz VisuMax femtosecond laser (Carl Zeiss Meditec AG, Jena, Germany) with a pulse energy of 150 nJ. The flap thickness was 120 μm. The optical zone diameter used was 8.0 mm, and the angle of the lenticular side cut was 90° (12 o'clock), with a width of 2.0 mm. For the FS-LASIK procedure, we used the 500-kHz VisuMax femtosecond laser to create a corneal flap and an Allegretto Wave Eye-Q refractive laser (Wavelight Laser Technologie AG, Erlangen, DE) to perform the ablation of the corneal stroma at a frequency of 400 Hz, with a pulse energy of 185 nJ. The flap thickness was 110 μm, and the optical zone diameter range was 6.5–7.1 mm. The duration of the intraoperative suction in FS-LASIK and SMILE was recorded as the hierarchical variables 1, 2, and 3, representing a duration of <10 s, between 10 and 20 s, and more than 20 s. The following medication was prescribed after surgery: topical steroids six times/day and gradually reduced over the following 30 days, topical antibiotics four times/day for seven days, and artificial tears four times/day for 30 days.

**Figure 1:**
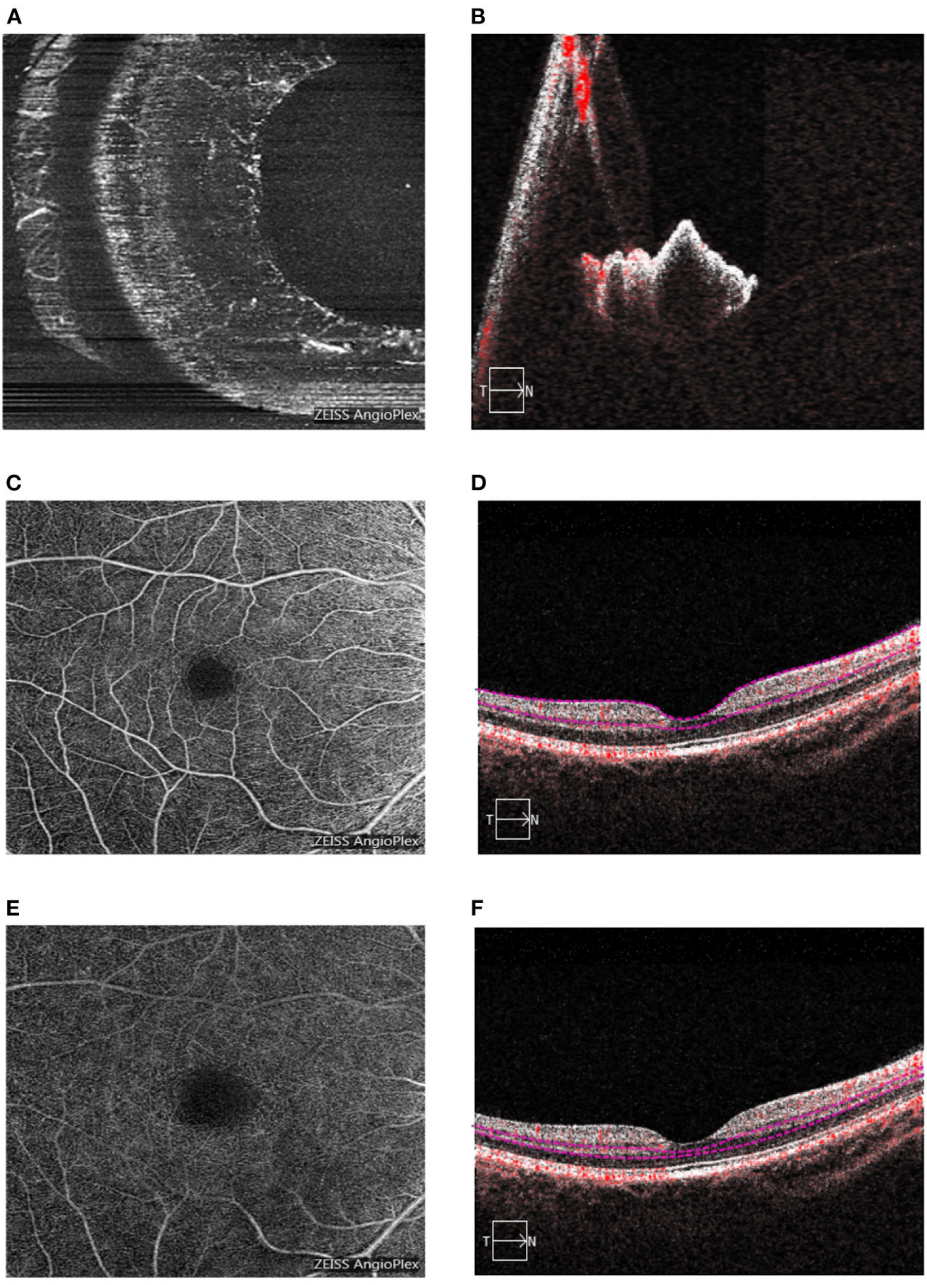
The iris and retina OCTA images.

OCTA was performed using the Cirrus HD-OCT 5000 system (Version 9.5.2, Carl Zeiss Meditec, Dublin, CA, USA). The iris OCTA images were acquired through a 3 × 3 mm^2^ scan (Figure 1). Quantitative detection of iris blood flow using OCTA images was performed as previously described (11). The specific acquisition and analysis method of the iris OCTA images was interpreted thoroughly, and the inter-visit repeatability was also verified in our last article (11). The retina OCTA images were acquired over a 6 × 6 mm^2^ scan. Segmentation of the superficial and deep capillary plexuses was automatically performed (Figure 1). Iris and retinal OCTA image quality control were conducted as follows. Firstly, the images were assessed by the Cirrus signal strength software after each acquisition, and it was considered “qualified” when a score of 8/10 was reached. Secondly, two independent observers evaluated the presence of vascular overlap or obvious artifacts, which made the image unsuitable for analysis. Thirdly, two independent investigators assessed and classified the iris OCTA images in three levels depending on vessel visibility. Images from participants showing different vessel visibility at different time points were considered “poor quality images.” Vessel area density (VAD) was calculated as the ratio between the area occupied by the vascular system and the total area in the binary angiogram. An increase in VAD was interpreted as vasodilation and increased blood flow. Vessel skeleton density (VSD) was calculated as the ratio between the length occupied by the vessels and the total area in the skeletal angiogram. A higher VSD value represented an increase in the number of vessels (12). Ophthalmic examinations were performed at 1 day pre-operatively and at 1 day, 1 week, 1 month, and 3 months post-operatively. The ophthalmic examination included: uncorrected distance visual acuity (UDVA), measured with an Early Treatment Diabetic Retinopathy Study (ETDRS) visual acuity chart (Precision Vision, Villa Park, Illinois, USA); autorefraction, measured using a KR-800 optometer (Topcon Corporation, Tokyo, Japan); central corneal thickness, measured with an SP-2000P specular microscope (Topcon Corporation, Tokyo, Japan); IOP, measured with a NIDEK-510 tonometer (Nidek Technologies, Gamagori, Japan); wavefront aberration, measured with a Pentacam (HR) system (WaveLight Allegro Oculyzer II, Erlangen, Germany); axial length (AL), mean corneal curvature, and white-to-white (WTW), all measured using an IOL Master 700 non-invasive optical biometer (Carl Zeiss Meditec AG, Jena, Germany). In addition, we examined the eyelids, tear river height, conjunctiva, cornea, lens, vitreous, and fundus using a Topcon SL-1E slit lamp microscope (Topcon Corporation, Tokyo, Japan) and an ophthalmoscope (66 Vision Tech Co., Ltd., Suzhou, Jiangsu, China).

The sample size was calculated using the Bioequivalence Program (Sample Size and Analysis) in StatsToDo (13). Considering α=0.05, the power of test was 80%. Based on our previous studies on iris VAD and VSD, the standard deviation (SD) was considered equal to 0.07, with a critical difference of 0.1. To obtain the 95% confidence interval (CI), the sample size required was 16 patients per group.

Results were expressed as the mean ± SD (standard deviation). Based on the Kolmogorov-Smirnov normality test results, a *t*-test or chi-squared test was used to compare basic characteristics between two groups. The generalized estimated equation method was used to analyze repeated measures of the iris and retinal blood flow indices and the relationship between these indices and other parameters. Post-operative VAD or VSD were considered the dependent variables, while the independent variables were sex ratio, age, pre-operative spherical equivalence (SE), IOP, mean corneal curvature, WTW, CCT, AL, pupil diameter (PD), group (FS-LASIK or SMILE), or intraoperative suction duration. A *p*-value lower than 0.05 was considered statistically significant. SAS 9.4 (SAS Institute, Inc., Cary, North Carolina, USA) software was used for statistical analysis.

## Results

Seventy-nine participants (79 eyes) were enrolled between July 2020 and September 2020. Of these, 27 eyes (34.17%) underwent FS-LASIK, and 52 eyes (65.83%) underwent SMILE. Baseline parameters are shown in Table 1. The SE and AL of the FS-LASIK group were significantly higher compared with the SMILE group (*p* < *0.05*). Mean SE and mean UDVA of all patients on post-operative day 1 were +0.66 ± 0.63 D and 0.97 ± 0.07 logMAR, respectively. The number of subjects at each follow-up visit is displayed in Table 2. The main cause of patients' dropouts after surgery was their reluctance to frequently visit the hospital during the COVID-19 pandemic. Baseline parameters of the participants who completed the post-operative follow-ups are also shown in Table 2. No significant difference was found when compared with the overall participants.

**Table 1:** Comparison of baseline parameters of patients in FS-LASIK and SMILE.

	**FS-LASIK** **(*N* = 27)**	**SMILE** **(*N* = 52)**	**Statistical** **value,** ***P*-value**
Male (number, %)	6, 22.22%	16, 30.77%	0.65^†^, 0.421
Age (year, mean± SD)	28.11 ± 7.58	28.42 ± 7.08	−0.18^#^, 0.856
SE (Diopter, mean ± SD)	−5.98 ± 1.78	−4.89 ± 1.46	−2.92^#^,0.004^*^
IOP (mmHg, mean ± SD)	16.21 ± 2.32	15.99 ± 2.57	0.35^#^, 0.724
Mean corneal curvature (Diopter, mean ± SD)	43.18 ± 1.41	43.59 ± 1.36	−1.25^#^, 0.215
WTW (mm, mean ± SD)	11.61 ± 0.45	11.60 ± 0.38	0.14^#^, 0.886
CCT (μm, mean ± SD)	532.28 ± 24.21	543.84 ± 21.24	−1.95^#^,0.057
PD (mm, mean ± SD)	6.86 ± 0.91	6.61 ± 0.80	0.79^#^, 0.384
AL (mm, mean ± SD)	25.83 ± 1.20	25.30 ± 0.85	2.05^#^, 0.045^*^
Suction time (represent case numbers and percentage of <10S, 10-20S, >20S)	4,14.81%; 14,51.85%; 9,33.33%	0,0.00%; 0,0.00%; 52,100.00%	44.89^†^, <0.000^*^

FS-LASIK, femtosecond laser in situ keratomileuses; SMILE, small-incision lenticule extraction; SD, standard deviation; SE, sepherical equivalence; IOP, intraocular pressure; WTW, white to white; CCT, central corneal thickness; PD, pupil diameter; AL, axial length.

# Denotes t-test.

† Denotes chi-square test.

* Denotes statistical significance.

**Table 2:** Comparison of baseline parameters of patients at different post-operative follow-up points with overall 79 cases.

	**Overall** **subjects** **(79 cases)**	**Subjects** **post-1 day** **(70 cases,** **88.6%)**	**Subjects** **post-1 day** **vs. baseline** **(statistical** **value, P)**	**Subjects** **post-7 days** **(77 cases,** **97.4%)**	**Subjects** **post-7 days** **vs. baseline** **(statistical** **value, P)**	**Subjects** **post-30** **days (68** **cases,** **84.8%)**	**Subjects** **post-30 days** **vs. baseline** **(statistical** **value, P)**	**Subjects** **post-90 days** **(54 cases,** **68.3%)**	**Subjects** **post-90 days** **vs. baseline** **(statistical** **value, P)**
Male (number, %)	22, 27.84%	20, 27.78%	0.00^†^, 0.964	21, 27.27%	0.50^†^, 0.479	18, 26.47%	0.46^†^, 0.497	12, 22.22%	2.69^†^, 0.101
Age (year, mean ± SD)	28.31 ± 7.21	28.15 ± 7.14	0.19^#^, 0.852	28.17 ± 7.24	0.17^#^, 0.864	28.53 ± 7.28	0.25^#^, 0.804	28.52 ± 6.96	0.22^#^, 0.826
Pre-Spherical equivalent (Diopter, mean ± SD)	−5.26 ± 1.65	−5.23 ± 1.72	0.14^#^,0.886	−5.27 ± 1.65	0.08^#^, 0.939	−5.27 ± 1.70	0.04^#^, 0.967	−5.31 ± 1.59	−0.24^#^, 0.809
IOP (mmHg, mean ± SD)	16.06 ± 2.47	15.93 ± 2.44	0.43^#^, 0.667	16.15 ± 2.45	0.31^#^, 0.759	16.07 ± 2.57	0.04^#^, 0.968	15.95 ± 2.52	0.31^#^, 0.755
Mean corneal curvature (Diopter, mean ± SD)	43.45 ± 1.38	43.46 ± 1.42	0.10^#^, 0.924	43.41 ± 1.38	0.19^#^, 0.848	43.45 ± 1.46	0.08^#^, 0.935	43.69 ± 1.36	1.31^#^, 0.195
WTW (mm, mean ± SD)	11.60 ± 0.37	11.61 ± 0.39	0.25^#^, 0.802	11.60 ± 0.37	0.00^#^, 1.000	11.61 ± 0.32	0.31^#^, 0.760	11.57 ± 0.32	0.55^#^, 0.584
Central corneal thickness (μm, mean ± SD)	539.25 ± 23.00	539.45 ± 22.10	0.07^#^, 0.947	539.37 ± 23.16	0.04^#^, 0.967	538.64 ± 23.44	0.19^#^, 0.851	538.46 ± 23.87	0.21^#^, 0.833
AL (mm, mean ± SD)	25.51 ± 1.03	25.49 ± 1.05	0.13^#^, 0.897	25.51 ± 1.04	0.01^#^, 0.993	25.53 ± 1.08	0.11^#^, 0.913	25.47 ± 0.94	0.27^#^, 0.789
Surgical methods (SMILE, %)	52, 65.82%	47, 65.28%	0.11^†^, 0.743	50, 64.94%	1.07^†^, 0.302	44, 64.71%	0.27^†^, 0.602	36, 66.67%	0.05^†^, 0.816
Signal strength	8.78 ± 0.68	8.56 ± 0.77	0.51^#^, 0.611	8.63 ± 0.94	0.35^#^, 0.727	8.73 ± 0.84	0.33^#^, 0.744	8.95 ± 0.68	−0.03^#^, 0.977
Suction time (represent case numbers and percentage of <10S, 10-20S, >20S)	4 (5.06%), 14 (17.72%), 61 (77.21%)	4 (5.56%), 12 (16.67%), 56 (77.78%)	0.93^†^, 0.627	4 (5.19%), 14 (18.18%), 59 (76.62%)	0.61^†^, 0.738	3 (4.41%), 13 (19.12%), 52 (76.47%)	0.98^†^, 0.612	2 (3.7%), 11 (20.37%), 41 (75.93)	1.33^†^, 0.512

SD, standard deviation; SE, sepherical equivalence; IOP, intraocular pressure; WTW, white to white; CCT, central corneal thickness; PD, pupil diameter; AL, axial length.

# Denotes t-test,

† Denotes chi-square test.

**Figure 2:**
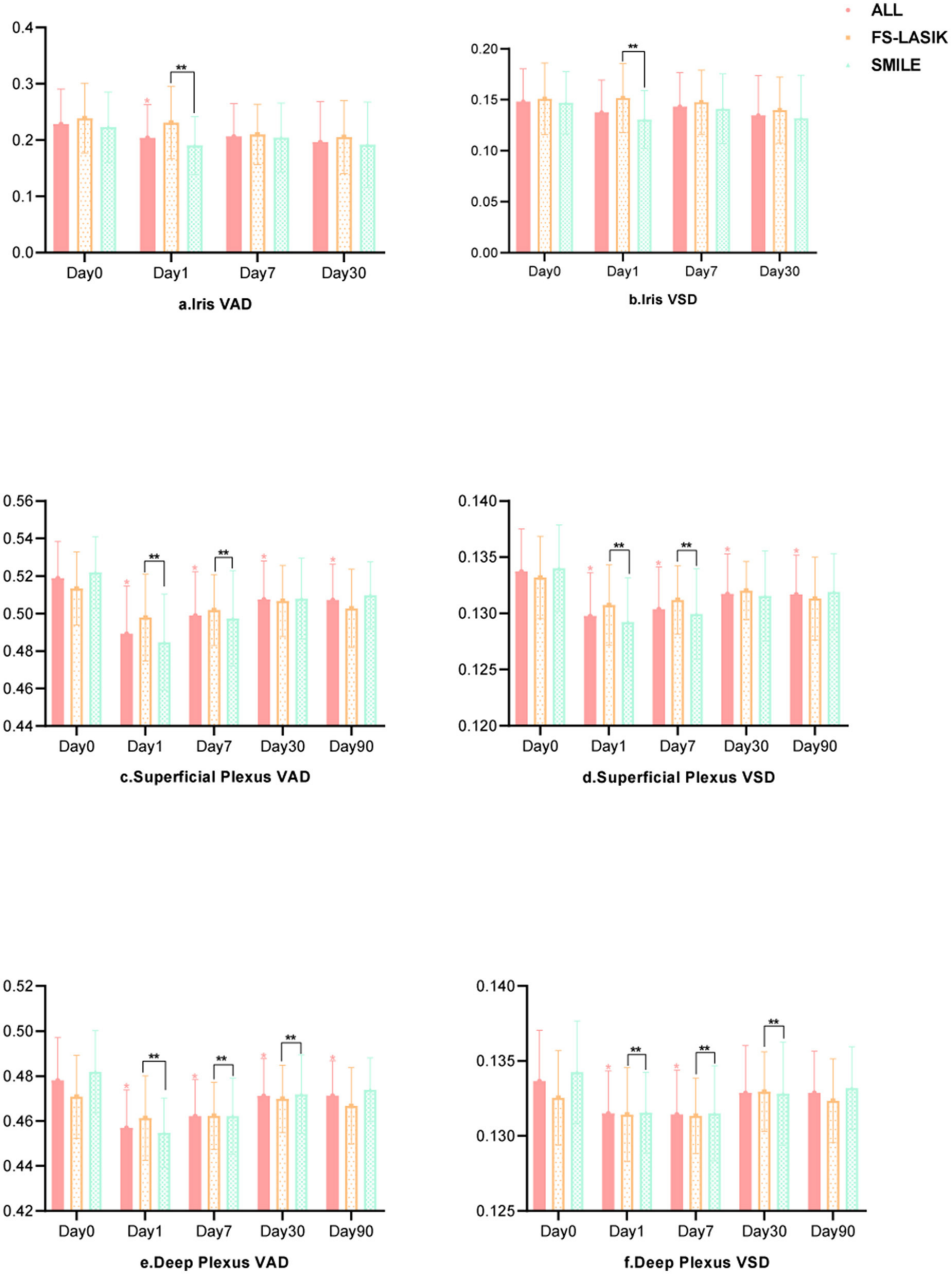
The postoperative changes of the iris and retina blood flow indices.

The iris and retinal VAD and VSD data of participants who completed the follow-up visits (days 1, 7, 30, and 90 after surgery) are shown in Table 3. The generalized estimated equation results are displayed in Table 4, including the influence of time, group, and other parameters. The blood flow indices in all patients, the FS-LASIK group, and the SMILE group, are shown in Figure 2. Statistically significant interactions between time and surgical procedure were observed.

**Table 3:** Iris and retinal VAD and VSD with time.

**Time**	**Group**	**Iris VAD**	**Iris VSD**	**Superficial** **plexus** **VAD**	**Superficial** **plexus** **VSD**	**Deep** **plexus** **VAD**	**Deep** **plexus** **VSD**
Pre-operatively	Overall subjects	0.231 ± 0.064	0.152 ± 0.034	0.518 ± 0.022	0.132 ± 0.001	0.478 ± 0.023	0.132 ± 0.001
	FS-LASIK	0.243 ± 0.058	0.154 ± 0.041	0.512 ± 0.023	0.132 ± 0.001	0.474 ± 0.018	0.132 ± 0.001
	SMILE	0.222 ± 0.062	0.151 ± 0.028	0.524 ± 0.022	0.131 ± 0.001	0.483 ± 0.022	0.131 ± 0.001
Post-1 day	Overall subjects	0.196 ± 0.058	0.144 ± 0.033	0.493 ± 0.034	0.130 ± 0.001	0.464 ± 0.024	0.132 ± 0.001
	FS-LASIK	0.231 ± 0.071	0.152 ± 0.032	0.503 ± 0.016	0.130 ± 0.001	0.463 ± 0.014	0.132 ± 0.001
	SMILE	0.193 ± 0.054	0.133 ± 0.032	0.483 ± 0.032	0.131 ± 0.001	0.458 ± 0.019	0.131 ± 0.001
Post-7 days	Overall subjects	0.211 ± 0.063	0.143 ± 0.029	0.498 ± 0.022	0.132 ± 0.001	0.463 ± 0.022	0.132 ± 0.001
	FS-LASIK	0.214 ± 0.049	0.150 ± 0.031	0.502 ± 0.020	0.130 ± 0.001	0.462 ± 0.018	0.132 ± 0.001
	SMILE	0.207 ± 0.060	0.140 ± 0.033	0.496 ± 0.032	0.132 ± 0.001	0.459 ± 0.024	0.132 ± 0.001
Post-30 days	Overall subjects	0.204 ± 0.071	0.134 ± 0.041	0.510 ± 0.021	0.130 ± 0.001	0.470 ± 0.018	0.131 ± 0.001
	FS-LASIK	0.213 ± 0.072	0.138 ± 0.027	0.509 ± 0.022	0.132 ± 0.001	0.469 ± 0.017	0.132 ± 0.001
	SMILE	0.194 ± 0.079	0.132 ± 0.038	0.511 ± 0.020	0.130 ± 0.001	0.470 ± 0.022	0.131 ± 0.001
Post-90 days	Overall subjects	/	/	0.513 ± 0.022	0.132 ± 0.001	0.470 ± 0.022	0.132 ± 0.001
	FS-LASIK	/	/	0.498 ± 0.021	0.132 ± 0.001	0.462 ± 0.019	0.130 ± 0.001
	SMILE	/	/	0.514 ± 0.022	0.133 ± 0.001	0.472 ± 0.012	0.132 ± 0.001

FS-LASIK, femtosecond laser in situ keratomileuses; SMILE, small-incision lenticule extraction; SD, standard deviation; VAD, vessel area density; VSD, vessel skeleton density.

**Table 4:** Analyses of the repeated measured iris and retinal blood flow indices and the relationship with other parameters.

**Parameter**	***Z***	***P***	**Parameter**	***Z***	***P***
**Iris VAD**			**Iris VSD**		
Intercept	0.22	0.826	Intercept	0.23	0.814
Day 1	−3.27	0.001	Day 1	−1.9	0.057
Day 7	0.33	0.741	Day 7	−0.81	0.415
Day 30	1.11	0.268	Day30	0.08	0.938
Group	−0.39	0.697	Group	−0.37	0.707
SE	0.53	0.599	SE	0.27	0.790
PD	3.21	0.001	PD	3.68	< 0.001
AL	0.5	0.618	AL	0.52	0.600
Suction time	0.15	0.879	Suction time	0.56	0.575
IOP	−0.86	0.389	IOP	−0.77	0.439
**Superficial plexus VAD**			**Superficial plexus VSD**		
Intercept	10.38	< 0.001	Intercept	13.5	< 0.001
Day 1	−8.54	< 0.001	Day 1	−7.54	< 0.001
Day 7	−5.69	< 0.001	Day 7	−6.05	< 0.001
Day 30	−4.02	< 0.001	Day 30	−4.17	< 0.001
Day 90	−4.02	< 0.001	Day 90	−4.11	< 0.001
Group	0.11	0.914	Group	−0.01	0.989
SE	−4.43	0.006	SE	−3.46	0.007
PD	−0.75	0.451	PD	−0.48	0.629
AL	−2.85	0.034	AL	−1.64	0.049
Suction time	−1.01	0.311	Suction time	−0.49	0.621
IOP	−0.25	0.804	IOP	−0.47	0.638
**Deep plexus VAD**			**Deep plexus VSD**		
Intercept	9	< 0.001	Intercept	10.92	< 0.001
Day 1	−7.68	< 0.001	Day 1	−5.32	< 0.001
Day 7	−6	< 0.001	Day 7	−5.43	< 0.001
Day 30	−2.68	0.007	Day 30	−1.86	0.062
Day 90	−2.79	0.005	Day 90	−1.93	0.053
Group	0.29	0.768	Group	0.41	0.683
SE	0.41	0.681	SE	0.44	0.656
PD	−0.95	0.342	PD	−0.37	0.709
AL	−0.89	0.374	AL	0.05	0.961
Suction time	−4.64	0.002	Suction time	−4.18	0.013
IOP	−0.33	0.737	IOP	−0.71	0.480

Dependent Variable: Iris VAD and VSD, retinal superficial plexus VAD and VSD, deep plexus VAD and VSD. Model: (Intercept), time, group, SE, PD, AL, suction time, IOP. (Parameters like CCT, sex, and age failed to reach statistically significant P-value and weren't shown here.) FS-LASIK was set to zero as the control group and SMILE was set to 1.

The iris VAD decreased on post-operative day 1 and recovered on day 7. On day 30 after surgery, no differences were found compared with baseline values. The iris VSD remained unchanged throughout the follow-up visits. For the iris circulation indices were stable at 30 days post-operatively, we didn't acquire the iris OCTA images on the last visit (day 90). The SMILE group showed significantly lower values of iris VAD and VSD on post-operative day 1 compared with the FS-LASIK group (*p* = 0.001, *p* = *0.057*). Both post-operative iris VAD and VSD were positively associated with the PD.

The VAD and VSD in the superficial capillary plexus dramatically decreased after the surgery and did not recover after 90 days. The SMILE group showed significantly lower VAD and VSD in the superficial capillary plexus on post-operative days 1 and 7 compared with the FS-LASIK group (all *p* < *0.000*). AL and SE were negatively associated with VAD and VSD in the superficial capillary plexus.

Similarly, the VAD in the deep capillary plexus significantly decreased after surgery and did not recover after 90 days. The VSD in the deep capillary plexus was lower than baseline on post-operative days 1 and 7, but no significant difference was observed on days 30 and 90. On days 1, 7, and 30 after surgery, the SMILE group displayed significantly lower values of VAD and VSD in the deep capillary plexus when compared with the FS-LASIK group (all *p* < *0.000*). Finally, suction duration was inversely correlated with VAD and VSD in the deep capillary plexus.

## Discussion

The results of the present study demonstrated that the iris blood flow indices decreased after FS-LASIK and SMILE surgeries but recovered on 7 days post-operatively. We found that the SMILE group was more vulnerable to iris blood flow changes than the FS-LASIK group. We also observed that the larger the PD, the better the post-operative iris blood flow. Regarding the retina, the VAD and VSD in the superficial and deep capillary plexuses significantly decreased after surgery and failed to recover even after 90 days. The SMILE group showed lower VAD and VSD values than the FS-LASIK group. The AL, SE, and suction duration negatively correlated with the post-operative VAD and VSD. The iris indices change after corneal refractive surgeries is not fully understood.

The negative pressure occurring during surgery is probably the main cause of ocular ischemia and hypoxia, which induce the post-operative decreased iris and macular vessel density. The suction ring-induced IOP elevation has been significantly reduced but not eliminated by updating surgical instruments such as the VisuMax femtosecond laser. When IOP is higher than the ophthalmic arterial pressure, a transient interruption of the blood flow can occur. Experiments in rabbits showed that the retinal blood flow self-regulation was disrupted when IOP was higher than 40 mmHg (14). Both FS-LASIK and SMILE groups experienced excessive IOP during surgery; therefore, a temporary interruption of the main ocular vessels increased the risk of transient ischemia. Interestingly, the suction duration was longer in SMILE than in FS-LASIK, which is in line with the former's worst blood flow indices. In summary, a series of hypoxic-ischemic events induced by intraoperative negative pressure may reduce post-operative blood flow. The femtosecond laser reaching the iris and retina may be another cause of decreased blood flow after surgery. The femtosecond laser has a wavelength of 1,053 nm and an excellent piercing ability. It penetrates the cornea and lens, but it may also affect deeper tissues such as the iris and retina, resulting in thermal and photochemical damage as well as vasoconstriction (8, 15). In particular, the thermal effect caused porcine iris pigment detachment and increased the iris temperature by 1–2°C (16). Furthermore, Hui Sun et al. reported a similar phenomenon in cadaveric retinas. In addition to the direct thermal effect, infrared light can change the energy state of the tissue, leading to subsequent thermochemical and photochemical reactions.

The surgical procedure was an influencing factor on postsurgical iris blood flow. We observed that the SMILE procedure caused more effect on the anterior segmental blood flow, probably due to the longer suction duration and the type of femtosecond laser used. Pre-operative IOP and suction duration were not influencing factors on iris indices, suggesting that the negative pressure was more relevant for the retina.

The role of the femtosecond laser in the iris damage is still a matter of debate. In a previous case report, the authors concluded that the iris atrophy shown after FS-LASIK resulted from elevated IOP-induced ischemic injury to the iris vessels (4). From our perspective, this damage was caused by the femtosecond laser itself because the iris lesion matched the laser trace. Unlike the retina, the iris presents an abundant blood supply and a better self-regulation (17). PD was positively correlated with the post-operative iris indices, indicating that larger pupils resulted in better postsurgical iris blood flow. We speculated that larger pupils reduced the laser damage during the procedure for lesser affected area (18). Interestingly, the iris VAD and VSD were not affected by patients' general characteristics (age, sex) or clinical parameters related to refractive status (SE, AL, mean corneal curvature, WTW, or CCT).

The trends in the postsurgical retinal indices in our study differed from those reported before (10, 19). Shen et al. divided the macular area into three zones: the foveal avascular zone (FAZ), the parafoveal area, and the perifoveal area. However, the exact way of dividing and measuring was not explained. The authors concluded that the vascular density returned to baseline values 1 week after SMILE in all areas except the FAZ zone (10). Using a similar division method, Gong et al. reported that 2 weeks after SMILE, the vascular density of the macular area returned to baseline levels, whereas superficial retinal vessel density (VD) in the parafoveal and perifoveal areas remained unchanged (20). Unlike our study, which used only one eye from each patient (independent sample), these two studies used data from both eyes. Therefore, the actual independent sample size was much lower than explained. Moreover, compared with these previous reports, we quantified a more extensive macular area, which gave us more comprehensive information about the retinal blood flow status.

Regarding the retina, the SMILE group showed a significant decrease in retinal blood flow compared with the FS-LASIK group. Participants in the FS-LASIK group presented higher SE and longer AL at baseline but no significant difference in pre-operative blood flow indices. Previous studies reported that patients with myopia showed reduced macular blood flow. This was attributed to elevated vascular resistance, narrowing, and fundus vessels stretching, as RE and AL increase, the situation becomes more serious (21). Curiously, in our study, the most affected patients belonged to the SMILE group, which showed lower SE and AL baseline values. Hence, we attributed these results to the longer femtosecond laser exposure and suction duration, which, in turn, elevated IOP intraoperatively and disrupted tissue perfusion. Differently, in the FS-LASIK procedure, the cornea and the lens absorbed most of the 193 nm excimer laser used to create the corneal flap, reducing its penetration to deeper tissues. In line with previous reports, we assumed that participants with higher SE and AL were more vulnerable to deficient ocular perfusion and femtosecond laser effects. Post-operative retinal blood flow indices were not associated with age, sex, mean corneal curvature, WTW, CCT, PD, or IOP. The age range of participants was relatively small in this study. Accordingly, altered retinal blood flow due to intraoperative ischemia and laser damage is generally independent of sex (20). Corneal curvature, WTW, and CCT may influence the refractive status after surgery, but their participation in intraoperative ischemic and laser damage is unlikely. Different from the iris, the PD did not prevent the laser from reaching the retina, probably due to the presence of the lens and vitreous body. Finally, pre-operative IOP was not associated with the intraoperative IOP.

We performed a quantitative OCTA examination of the iris to analyze the blood flow status. Inevitably, OCTA images of the iris presenting more pigmentation (e.g., Asian patients) showed less vasculature information. However, this was not a limitation when analyzing the differences between pre-operative and post-operative iris blood flow indices. A possible limitation of our study was that the surgical procedures were not randomly selected, resulting in significant differences in pre-operative AL and SE between the two groups.

In summary, FS-LASIK and SMILE surgeries significantly reduced the iris and retinal blood flow in the short run, although patients finally showed full visual recovery. Thus, patients prone to anterior segment lesions should be carefully considered before undergoing these procedures. In addition, iris blood flow changes should be evaluated after other surgical procedures (e.g., cataract surgery) or diseases (e.g., glaucoma). The specific mechanisms underlying the reduced blood flow after corneal refractive surgeries need further elucidation. Longer follow-ups and advanced imaging techniques may help reveal more relevant features of iris blood flow.

## Data availability statement

The original contributions presented in the study are included in the article/supplementary material, further inquiries can be directed to the corresponding author/s.

## Ethics statement

The studies involving human participants were reviewed and approved by Shanghai General Hospital. The patients/participants provided their written informed consent to participate in this study.

## Author contributions

LC contributed to the data acquisition, analysis, and manuscript editing. WenwX, SL, and LZ contributed to the concept. HZ contributed to the concept, design, definition of intellectual content, and manuscript review. XH, WY, WenX, and YW participated in the procedure and data collection. All authors contributed to the article and approved the submitted version.

## Funding

This study was funded by the Project of Shanghai Shen Kang Hospital Development Centre (Grant Nos. SHDC2020CR30538, SHDC2018110, and SHDC12018X16), Shanghai Engineering Research Center for Precise Diagnosis and Treatment of Eye Diseases (Project No. 19DZ2250100), the Science and Technology Commission of Shanghai Municipality (Project No. 20DZ1100200), Shanghai Municipal Commission of Health (public health system three-year plan-Key Subjects) (Project No. GWV10.1-XK7), and Shanghai Municipal Health Commission (Grant No. SHDC20194Y0441).

## Conflict of interest

The authors declare that the research was conducted in the absence of any commercial or financial relationships that could be construed as a potential conflict of interest.

## Publisher's note

All claims expressed in this article are solely those of the authors and do not necessarily represent those of their affiliated organizations, or those of the publisher, the editors and the reviewers. Any product that may be evaluated in this article, or claim that may be made by its manufacturer, is not guaranteed or endorsed by the publisher.
